# [Benz­yl(2-pyridyl­methyl­idene)amine]­dichloridomercury(II)

**DOI:** 10.1107/S1600536810035889

**Published:** 2010-09-11

**Authors:** Young-Inn Kim, Sung Kwon Kang

**Affiliations:** a Department of Chemistry Education and Interdisciplinary Program of Advanced Information and Display Materials, Pusan National University, Busan 609-735, Republic of Korea; b Department of Chemistry, Chungnam National University, Daejeon 305-764, Republic of Korea

## Abstract

The Hg^II^ ion in the title complex, [HgCl_2_(C_13_H_12_N_2_)], adopts a distorted tetra­hedral geometry being coordinated by two Cl anions and by two N atoms of the benz­yl(2-pyridyl­methyl­ene)amine ligand. The Cl—Hg—Cl plane is twisted at 70.1 (1)° from the mean plane of the chelate ring. In the crystal structure, inter­molecular π–π inter­actions [centroid–centroid distance = 3.793 (3) Å] between the aromatic rings link the mol­ecules into zigzag chains extending along [010].

## Related literature

For chemosensors of mercury ions, see: Zhou *et al.* (2010 ▶). For electroluminescent devices, see: Fan *et al.* (2009 ▶). For the crystal structures and luminescence of related Hg complexes, see: Kim *et al.* (2008 ▶, 2010 ▶); Seo *et al.* (2009*a* ▶,*b* ▶).
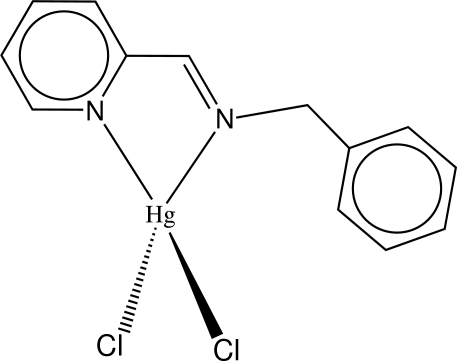


## Experimental

### 

#### Crystal data

[HgCl_2_(C_13_H_12_N_2_)]
*M* *_r_* = 467.74
Monoclinic, 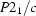

*a* = 8.2736 (1) Å
*b* = 11.8828 (2) Å
*c* = 14.1191 (2) Å
β = 94.343 (1)°
*V* = 1384.11 (3) Å^3^
*Z* = 4
Mo *K*α radiation
μ = 11.49 mm^−1^
*T* = 295 K
0.22 × 0.20 × 0.18 mm

#### Data collection

Bruker SMART CCD area-detector diffractometer
Absorption correction: multi-scan (*SADABS*; Bruker, 2002 ▶) *T*_min_ = 0.094, *T*_max_ = 0.118
14227 measured reflections
3432 independent reflections
2797 reflections with *I* > 2σ(*I*)
*R*_int_ = 0.026

#### Refinement

*R*[*F* ^2^ > 2σ(*F* ^2^)] = 0.026
*wR*(*F* ^2^) = 0.056
*S* = 1.03
3432 reflections
163 parameters
H-atom parameters constrained
Δρ_max_ = 1.03 e Å^−3^
Δρ_min_ = −1.61 e Å^−3^

### 

Data collection: *SMART* (Bruker, 2002 ▶); cell refinement: *SAINT* (Bruker, 2002 ▶); data reduction: *SAINT*; program(s) used to solve structure: *SHELXS97* (Sheldrick, 2008 ▶); program(s) used to refine structure: *SHELXL97* (Sheldrick, 2008 ▶); molecular graphics: *ORTEP-3 for Windows* (Farrugia, 1997 ▶) and *DIAMOND* (Brandenburg, 2010 ▶); software used to prepare material for publication: *WinGX* (Farrugia, 1999 ▶).

## Supplementary Material

Crystal structure: contains datablocks global, I. DOI: 10.1107/S1600536810035889/cv2760sup1.cif

Structure factors: contains datablocks I. DOI: 10.1107/S1600536810035889/cv2760Isup2.hkl

Additional supplementary materials:  crystallographic information; 3D view; checkCIF report

## Figures and Tables

**Fig. 1.**
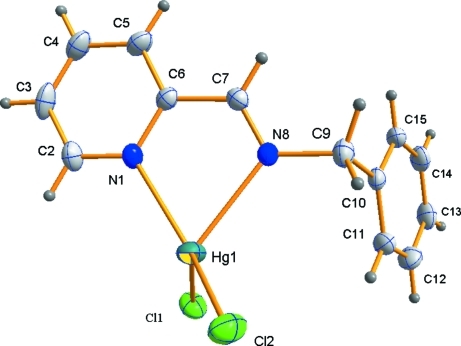
Molecular structure of (I), showing the atom-numbering scheme and 30% probability displacement ellipsoids.

**Fig. 2.**
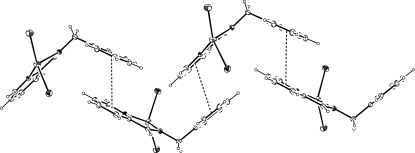
A portion of the crystal packing showing zigzag chain (extended in direction [010]) of the molecules linked by π-π interactions (dotted lines).

## supplementary crystallographic information

### Comment

Luminescent mercury(II) compounds with nitrogen-containing ligands have reported in studies concerning their performance in chemosensors for mercury ions (Zhou *et al.*, 2010) and electroluminescent devices (Fan *et al.*, 2009). As an extension of our work (Kim *et al.*, 2010; Seo *et al.*, 2009*a*, *b*; Kim *et al.*, 2008) on luminescent mercury(II) complexes, herein, we report here the crystal structure and  luminescent properties of the title Hg^II^ chloride complex with benzyl(2-pyridylmethylene)amine (bpma), (I).

In (I) (Fig. 1), the Hg^II^ ion is coordinated by two N atoms of bpma ligand and two Cl anions. The angles around Hg atom are in the range of 71.00 (10) – 136.35 (8)°, suggesting the coordination geometry around the Hg atom is described as a distorted tetrahedron. The Cl—Hg—Cl plane is twisted at 70.1 (1)° from the mean plane of the chelate ring. The phenyl ring on the bpma ligand is twisted out of the pyridine plane, and form a dihedral angel of 67.9 (1)°. In the crystal structure, there are weak π-π interactions between the aromatic rings of the discrete units (Table 1), which link the molecules into zigzag chains extended in direction [010] (Fig. 2).

The title complex exhibited an emission (λ_max,PL_ = 426 nm in DMF) upon 280 nm excitation with the quantum yield of 2.9%, which was contributed from the intra-ligand (IL) ^1^(π-π^*^) transition.

### Experimental

All of the reagents and solvents were commercially purchased from Aldrich and used without further purification. Benzyl(2-pyridylmethylene)amine (bpma) was synthesized from the reaction of 2-pyridinecarboxylaldehyde and benzylamine. A solution of benzylamine (20 mmol) in methanol (30 ml) was added to a solution of 2-pyridinecarboxylaldehyde (20 mmol) in methanol (30 ml), and the mixture was stirred for 3 h at room temperature. To a stirred solution of bpma was added mercuric chloride (20 mmol) in methanol (30 ml). The solution was stirred for 6 h at room temperature. The white crystals were obtained after recrystallization from methanol solution.

### Refinement

All H atoms were positioned geometrically and refined using a riding model, with C—H = 0.93 - 0.97 Å, and with *U*_iso_(H) = 1.2*U*_eq_(C). The maximal residual peak and minimal residual hole situated at 0.78 and 0.79 Å, respectively, from the Hg1 atom.

### Crystal data

**Table:**

[HgCl_2_(C_13_H_12_N_2_)]	*F*(000) = 872
*M**_r_* = 467.74	*D*_x_ = 2.245 Mg m^−^^3^
Monoclinic, *P*2_1_/*c*	Mo *K*α radiation, λ = 0.71073 Å
Hall symbol: -P 2ybc	Cell parameters from 5055 reflections
*a* = 8.2736 (1) Å	θ = 2.2–27.7°
*b* = 11.8828 (2) Å	µ = 11.49 mm^−^^1^
*c* = 14.1191 (2) Å	*T* = 295 K
β = 94.343 (1)°	Block, colourless
*V* = 1384.11 (3) Å^3^	0.22 × 0.2 × 0.18 mm
*Z* = 4	

### Data collection

**Table:**

Bruker SMART CCD area-detector diffractometer	2797 reflections with *I* > 2σ(*I*)
φ and ω scans	*R*_int_ = 0.026
Absorption correction: multi-scan (*SADABS*; Bruker, 2002)	θ_max_ = 28.3°, θ_min_ = 2.2°
*T*_min_ = 0.094, *T*_max_ = 0.118	*h* = −11→11
14227 measured reflections	*k* = −15→15
3432 independent reflections	*l* = −18→18

### Refinement

**Table:**

Refinement on *F*^2^	0 restraints
Least-squares matrix: full	H-atom parameters constrained
*R*[*F*^2^ > 2σ(*F*^2^)] = 0.026	*w* = 1/[σ^2^(*F*_o_^2^) + (0.0212*P*)^2^ + 1.9423*P*] where *P* = (*F*_o_^2^ + 2*F*_c_^2^)/3
*wR*(*F*^2^) = 0.056	(Δ/σ)_max_ = 0.001
*S* = 1.03	Δρ_max_ = 1.03 e Å^−^^3^
3432 reflections	Δρ_min_ = −1.61 e Å^−^^3^
163 parameters	

### Special details

**Table:**

Geometry. All e.s.d.'s (except the e.s.d. in the dihedral angle between two l.s. planes) are estimated using the full covariance matrix. The cell e.s.d.'s are taken into account individually in the estimation of e.s.d.'s in distances, angles and torsion angles; correlations between e.s.d.'s in cell parameters are only used when they are defined by crystal symmetry. An approximate (isotropic) treatment of cell e.s.d.'s is used for estimating e.s.d.'s involving l.s. planes.

### Fractional atomic coordinates and isotropic or equivalent isotropic displacement parameters (Å^2^)

**Table:**

	*x*	*y*	*z*	*U*_iso_*/*U*_eq_	
Hg1	0.16420 (2)	0.368809 (15)	0.072427 (10)	0.04946 (7)	
N1	0.2879 (3)	0.4688 (2)	0.1992 (2)	0.0354 (7)	
C2	0.3837 (5)	0.5579 (3)	0.1919 (3)	0.0457 (9)	
H2	0.4039	0.5842	0.1319	0.055*	
C3	0.4543 (5)	0.6126 (3)	0.2713 (4)	0.0561 (12)	
H3	0.5191	0.6756	0.2645	0.067*	
C4	0.4283 (5)	0.5735 (4)	0.3594 (4)	0.0539 (11)	
H4	0.474	0.6096	0.4133	0.065*	
C5	0.3333 (5)	0.4797 (4)	0.3671 (3)	0.0471 (10)	
H5	0.3157	0.4506	0.4266	0.056*	
C6	0.2642 (4)	0.4290 (3)	0.2860 (2)	0.0341 (7)	
C7	0.1618 (4)	0.3282 (3)	0.2917 (3)	0.0353 (8)	
H7	0.1324	0.3039	0.3507	0.042*	
N8	0.1135 (4)	0.2744 (2)	0.2183 (2)	0.0350 (6)	
C9	0.0108 (5)	0.1746 (3)	0.2275 (3)	0.0430 (9)	
H9A	−0.0839	0.1799	0.1828	0.052*	
H9B	−0.0258	0.1717	0.2911	0.052*	
C10	0.1029 (4)	0.0685 (3)	0.2085 (3)	0.0356 (8)	
C11	0.1158 (5)	0.0304 (3)	0.1168 (3)	0.0441 (9)	
H11	0.0678	0.0707	0.0657	0.053*	
C12	0.1996 (5)	−0.0669 (4)	0.1011 (3)	0.0519 (10)	
H12	0.2082	−0.0917	0.0392	0.062*	
C13	0.2707 (6)	−0.1275 (3)	0.1754 (4)	0.0520 (10)	
H13	0.3261	−0.1937	0.164	0.062*	
C14	0.2598 (5)	−0.0905 (4)	0.2664 (3)	0.0517 (10)	
H14	0.3086	−0.1312	0.3171	0.062*	
C15	0.1764 (5)	0.0073 (3)	0.2832 (3)	0.0442 (9)	
H15	0.1696	0.0323	0.3452	0.053*	
Cl1	0.39293 (15)	0.30109 (10)	−0.00900 (8)	0.0599 (3)	
Cl2	−0.10125 (14)	0.35992 (11)	−0.00837 (8)	0.0607 (3)	

### Atomic displacement parameters (Å^2^)

**Table:**

	*U*^11^	*U*^22^	*U*^33^	*U*^12^	*U*^13^	*U*^23^
Hg1	0.05046 (10)	0.06449 (12)	0.03322 (8)	−0.00618 (8)	0.00188 (6)	−0.00268 (7)
N1	0.0322 (15)	0.0313 (16)	0.0423 (16)	0.0000 (12)	−0.0001 (12)	0.0043 (13)
C2	0.037 (2)	0.037 (2)	0.063 (3)	−0.0032 (17)	0.0030 (18)	0.0095 (19)
C3	0.034 (2)	0.029 (2)	0.102 (4)	−0.0029 (16)	−0.009 (2)	−0.003 (2)
C4	0.052 (2)	0.042 (2)	0.065 (3)	0.003 (2)	−0.016 (2)	−0.014 (2)
C5	0.049 (2)	0.046 (2)	0.044 (2)	0.0038 (19)	−0.0083 (18)	−0.0101 (18)
C6	0.0327 (17)	0.0312 (18)	0.0380 (18)	0.0056 (15)	−0.0002 (14)	−0.0026 (15)
C7	0.0371 (19)	0.0343 (18)	0.0351 (18)	0.0044 (15)	0.0070 (14)	0.0033 (15)
N8	0.0356 (15)	0.0318 (16)	0.0379 (16)	−0.0032 (13)	0.0044 (12)	0.0003 (13)
C9	0.039 (2)	0.037 (2)	0.055 (2)	−0.0078 (17)	0.0115 (17)	−0.0017 (18)
C10	0.0355 (18)	0.0303 (19)	0.0414 (19)	−0.0094 (15)	0.0058 (15)	−0.0001 (15)
C11	0.054 (2)	0.038 (2)	0.039 (2)	−0.0025 (18)	−0.0003 (17)	0.0024 (17)
C12	0.059 (3)	0.047 (2)	0.050 (2)	−0.002 (2)	0.009 (2)	−0.012 (2)
C13	0.055 (2)	0.031 (2)	0.072 (3)	0.0005 (19)	0.012 (2)	0.003 (2)
C14	0.055 (3)	0.040 (2)	0.059 (3)	−0.005 (2)	−0.003 (2)	0.014 (2)
C15	0.056 (2)	0.042 (2)	0.0353 (19)	−0.0159 (19)	0.0032 (17)	0.0016 (17)
Cl1	0.0642 (7)	0.0605 (7)	0.0570 (6)	0.0117 (6)	0.0186 (5)	0.0063 (5)
Cl2	0.0559 (6)	0.0762 (8)	0.0479 (6)	−0.0078 (6)	−0.0095 (5)	−0.0080 (5)

### Geometric parameters (Å, °)

**Table:**

Hg1—N1	2.321 (3)	C7—H7	0.93
Hg1—Cl2	2.3993 (11)	N8—C9	1.470 (5)
Hg1—N8	2.409 (3)	C9—C10	1.507 (5)
Hg1—Cl1	2.4249 (11)	C9—H9A	0.97
N1—C2	1.331 (5)	C9—H9B	0.97
N1—C6	1.342 (4)	C10—C11	1.382 (5)
C2—C3	1.387 (6)	C10—C15	1.384 (5)
C2—H2	0.93	C11—C12	1.375 (6)
C3—C4	1.359 (7)	C11—H11	0.93
C3—H3	0.93	C12—C13	1.368 (6)
C4—C5	1.373 (6)	C12—H12	0.93
C4—H4	0.93	C13—C14	1.368 (6)
C5—C6	1.379 (5)	C13—H13	0.93
C5—H5	0.93	C14—C15	1.381 (6)
C6—C7	1.473 (5)	C14—H14	0.93
C7—N8	1.258 (5)	C15—H15	0.93
			
Cg1···Cg2^i^	3.793 (3)		
			
N1—Hg1—Cl2	136.35 (8)	C7—N8—C9	119.1 (3)
N1—Hg1—N8	71.00 (10)	C7—N8—Hg1	113.8 (2)
Cl2—Hg1—N8	99.99 (8)	C9—N8—Hg1	126.2 (2)
N1—Hg1—Cl1	102.78 (8)	N8—C9—C10	110.9 (3)
Cl2—Hg1—Cl1	118.63 (4)	N8—C9—H9A	109.5
N8—Hg1—Cl1	116.32 (8)	C10—C9—H9A	109.5
C2—N1—C6	118.7 (3)	N8—C9—H9B	109.5
C2—N1—Hg1	125.3 (3)	C10—C9—H9B	109.5
C6—N1—Hg1	115.9 (2)	H9A—C9—H9B	108.1
N1—C2—C3	121.8 (4)	C11—C10—C15	118.7 (4)
N1—C2—H2	119.1	C11—C10—C9	121.1 (4)
C3—C2—H2	119.1	C15—C10—C9	120.2 (3)
C4—C3—C2	119.6 (4)	C12—C11—C10	120.2 (4)
C4—C3—H3	120.2	C12—C11—H11	119.9
C2—C3—H3	120.2	C10—C11—H11	119.9
C3—C4—C5	118.8 (4)	C13—C12—C11	120.8 (4)
C3—C4—H4	120.6	C13—C12—H12	119.6
C5—C4—H4	120.6	C11—C12—H12	119.6
C4—C5—C6	119.5 (4)	C14—C13—C12	119.7 (4)
C4—C5—H5	120.3	C14—C13—H13	120.1
C6—C5—H5	120.3	C12—C13—H13	120.1
N1—C6—C5	121.6 (4)	C13—C14—C15	120.1 (4)
N1—C6—C7	117.5 (3)	C13—C14—H14	120
C5—C6—C7	120.9 (3)	C15—C14—H14	120
N8—C7—C6	121.1 (3)	C14—C15—C10	120.5 (4)
N8—C7—H7	119.5	C14—C15—H15	119.7
C6—C7—H7	119.5	C10—C15—H15	119.7

Symmetry codes: (i) −*x*+1, *y*+1/2, −*z*+1/2.
